# Prevalence of depressive disorders in Rasht, Iran: A community based study

**DOI:** 10.1186/1745-0179-4-20

**Published:** 2008-07-04

**Authors:** Mohamad Jafar Modabernia, Hossein Shodjai Tehrani, Mahnaz Fallahi, Maryam Shirazi, Amir Hossein Modabbernia

**Affiliations:** 1 Department of Psychiatry, Guilan University of Medical Sciences, Rasht, Iran; 2 Deputy of Research, Guilan University of Medical Sciences, Rasht, Iran; 3 Tehran University of Medical Sciences, Tehran, Iran

## Abstract

**Introduction:**

Depression is a well known health problem worldwide. Prevalence of depressive disorders varies in different societies.

**Aim:**

to determine the prevalence of depressive disorders and some associated factors in Rasht City (Northern part of Iran).

**Materials and methods:**

4020 subjects were selected among 394925 residents of Rasht aged between 18–70 during 2003 – 2004. In the first phase, subjects were screened by Beck's Depression Inventory. In the second phase, those who scored more than 15 were assessed through semi-structured psychiatric interview (DSMIV-TR). Socio-demographic characteristics including age, gender, marital status, educational level, and socio-economic class were recorded as well.

**Results:**

9.5% of samples (63% female and 37% male) were diagnosed by depressive disorders. The prevalence of minor depressive disorder, dysthymia and major depressive disorder was 5%, 2/5%, and 1% respectively. Socio-economic class was significantly associated with both depressive symptoms based on BDI score (p < 0.001) and depressive disorders based on clinical interview (p < 0.001).

**Conclusion:**

Comparing to other studies, this study revealed that prevalence of dysthymic and minor depressive disorder were more than major depressive disorder, and low socio-economic class was the most significant risk factor associated with depression. Regarding our study limitations, researchers and policy makers should not consider our findings as conclusive results. Findings of this study could be applied by researchers using analytical methodology to assess relationship between depressive disorders and associated factors.

## Background

Depression has been ranked as the forth most urgent health problem in the world by World Health Organization (WHO) in 2005. The disability caused by depression is comparable or even more than those caused by chronic pain, hypertension, diabetes mellitus and coronary artery disease. Suicide attempt occurs in approximately 15% of depressive patients, specially young and elderly men [1].

Prevalence of depressive disorders varies in different socio-cultural populations. In a study on depressive disorders in 8764 randomly selected samples of general population in five European countries, the prevalence of depressive disorders was assessed by a cross-sectional two-phase community study using Beck's Depression inventory in Phase 1 and Schedule for Clinical Assessment in Neuropsychiatry in Phase 2. The Overall prevalence of depression was 8.56%. The weighted prevalence of different diagnostic categories of depressive disorders was as follows; major depressive episode: 6.7%, dysthymia: 1% and adjustment disorder: 0.5% [2].

The prevalence of psychiatric disorders in 2979 individuals was estimated by Composite International Diagnostic Interview in Chili. The one-month prevalence of depressive disorders was 6.3% (3.4% major depressive disorder and 2.9% dysthymia). Those aged 45–54 years, singles and separated individuals, people with low incomes and medium level of education were at increased risk of depressive disorders [3].

In a cross-sectional population-based epidemiological study in Iran, 25180 individuals who aged more than 18 were assessed using Schedule for Affective Disorders and Schizophrenia (SADS). The current prevalence of depressive disorders was 2% (1.8% major depressive disorder and 0.2% minor depressive disorder) and it was associated with female gender, lower education, being married, being middle-aged, living in cities and not being a homemaker [4].

Noorbala et al studied 879 individuals to estimate the prevalence of psychiatric disorders in Tehran (the capital city of Iran). Using DSMIV criteria and clinical interview, he concluded that the prevalence of depressive disorders was 9.2% (4.4% major depressive disorders, 3.9% dysthymia and 0.8% minor depressive disorder) [5].

Most of the literatures show that the gender ratio (F: M) in depressive disorders is between 1.5:1 to 2:1 [2-4]; however, in one study, minimal gender differences was found [6]. Those who aged 45–54 and 80–82 are most likely to develop depressive disorders [3,6]. In a study in Iran, the highest prevalence of depressive disorders was found in 41–55 year-old age group [4]. Review of literature shows that unemployment, medium educational level, elderly, being single, divorced and separated as well as low income are associated with depressive disorders [3,6,7]. In one study in Iran, depression was more prevalent in married people [4]. Depressive disorders were found to be unrelated to educational level in some literatures [8]; however, others showed higher prevalence rates in people with low educational levels [3,4] or high-school education [7]. The relationship between socio-economic situation and depressive disorders has remained subject of question. Vicente demonstrated that depressive disorders were more common in low-income people [3]. By contrast, a higher rate of depressive disorders in employed individuals was reported in another study [4]. Nevertheless, the relationship between socio-economic class and depressive disorders is controversial [9].

Although several epidemiologic studies on the prevalence of depressive disorders have been carried out in Iran, their opposed results [4,5,7], indefinite definitions of socio-economic class and diagnostic criteria in addition to the differences in sample size and cultural situation make it difficult to generalize their findings to Guilan society. Regarding the aforementioned reasons and the unclear epidemiology of depressive disorders in Guilan province, this study was performed to estimate the prevalence of depressive disorders and some associated factors in Rasht City (capital city of Guilan province located in Northern part of Iran).

## Methods

A descriptive study was performed on 18–70 year-old Rasht residents from January 2003 to December 2004. Regarding previous studies, the prevalence of depression was considered 5% [5,10]. Based on precision of 1% and confidence level of 95%, 4020 subjects out of a total number of 394925 residents of Rasht were selected by multistage cluster sampling method. 201 clusters were chosen with 20 subjects in each of them and they were proportionally distributed between health centers. At first, the list of Rasht health centers was obtained from Vice-Chancellorship for health of Guilan University of Medical Sciences. The number of households in each health center was calculated and clusters were randomly chosen from households of 15 urban and 15 rural health centers. Then, some households were randomly selected between households of each cluster and eventually, one subject was chosen in each household randomly. Male or female subjects were not selected separately. Selecting the location of the first cluster was based on a random number table. Next, using a systematic sampling technique knowing the inter-cluster interval, subsequent clusters were selected.

Individuals with any communication problem, mental retardation, chronic schizophrenia, and those who did not respond for any reason were excluded.

### Data collection tools and assessment

The assessment was performed in two phases. In the first phase, possible cases of depressive disorders were identified using Beck's Depression Inventory (BDI). Clinical psychologists-after receiving an initial training course on the goals of the study-referred to selected houses and invited subjects to participate in the research. In the case of subject's refusal to participate in the study, another person form the same house was selected randomly. Subjects who were not available, were contacted weekly up to one month and eventually if they were not found, a person from neighbor house was replaced by them. Finally, 4020 subjects were participated in the study while 504 subjects refused to complete the questionnaire and response rate was 89%.

BDI is a 21-item standard self report questionnaire, evaluating sad mood, pessimistic outlook, feelings of guilt and loss of appetite and is defined as follows: symptom-free (0–15), mild depression (16–30), moderate depression (31–46) and sever depression (47–63) We applied the Persian version of BDI which was validated by Kaviani et al [11].

Subjects filled in BDI within 15 minutes. In illiterates, the questions were read for them by research workers and they completed the questionnaires with regard to subjects' responses. Every eight questionnaires were rechecked by a clinical psychologist with respect to a prepared check list.

BDI was accompanied with a questionnaire on demographic data (age, gender, socioeconomic class, educational level and marital status). Socio-economic class was divided into 3 classes: (first, second, and third) [12]. Subjects' age was grouped in 3 categories: (15–30), (31–40) and over 40. Marital status was defined as single, married and others (divorced, widow, widower). Regarding educational level, subjects were categorized in 4 different groups: ("illiterate/elementary school", "guidance/high-school", "diploma/associate degrees", "bachelor/higher").

In the second phase, all of those scoring over 15 were assessed through a semi-structured interview by a psychiatrist to identify depressive criteria according to (DSMIV-TR) including major depressive disorder, minor depressive disorder and dysthymia.

We used descriptive statistics to show frequency rate of variables. Chi-square test was used for categorical variables, Kappa for determination of agreement coefficient between Beck's score and depressive disorders, and Mantel Hanzel Chi-square for comparison of different levels of depressive symptoms and depressive disorders in major subgroups of variables. p < 0.05 was considered as the significant level.

All the family charts, statistical tests and questionnaires are available from the authors upon request.

## Results

Table 1 demonstrates the demographic characteristics of our subjects.

**Table 1:** The demographic characteristics of under study sample

Variables	No (%) (n = 4020)
**Gender**	
female	2523(63.05)
male	1485(36.94)

**Socio-economic class (score)**	
high-class (19–25)	19(0.47)
middle-class (12–18)	670(16.66)
low-class (5–11)	3331(82.86)

**Marital status**	
single	1478(36.76)
married	2403(59.77)
others*	139(3.64)

**Educational level**	
illiterate/elementary school	618(15.37)
guidance/high-school	1143(28.43)
diploma/associate degrees	1772(44.07)
bachelor/higher	487(12.1)

**Age (yrs)**	
15–30	2117(58.65)
31–39	840(20.39)
over 40	1083(26.93)

*others: divorced, widow, widower.

Response rate in the first phase of the study was 89%. In BDI, 23% of subjects scored more than 15; however, none of the samples who scored 15–19 had depressive disorders in clinical interview. 88 out of 700 subjects, who scored more than 19, were interviewed by a psychiatric (response rate = 13%). We used kappa in order to determine the agreement coefficient between BDI score and psychiatric interview result to evaluate different types of depressive disorders. It was calculated 0.404 for minor depressive disorder and Beck's score between 20 and 30, 0.609 for dysthymic disorder and Beck's score between 31 and 46, 0.876 for major depressive disorder and Beck's score ≥ 47, and 0.556 for total depressive disorders diagnosed by clinical interview and depressive symptoms found in BDI.

9.7% of subjects were diagnosed by depressive disorders and the prevalence of minor depressive disorder, dysthymia and major depressive disorder was 5%, 2.5% and 1% respectively (Table 2).

**Table 2:** The prevalence of depressive disorders according to studied variables

Agreement coefficient	0.404	0.609	0.556	0.876
**Variables/depressive disorders**	Minor no(%) 96(5%)	Dysthymia no(%) 155(2.5%)	Major no(%) 49(1%)	Total no(%) 700(9.7%)

**Gender**				
female	329(3.30)	96(1.45)	36(0.78)	461(6.37)
male	167(1.67)	59(0.89)	13(0.28)	239(3.30)

**Socio-economic class (score)**				
high-class (19–25)	1(0.01)	1(0.01)	0 (0)	2(0.02)
middle-class (12–18)	68(0.68)	15(0.22)	4(0.87)	87(1.20)
low-class (5–11)	427(4.29)	139(2.10)	45(0.98)	611(8.45)

**Marital status**				
single	197(1.97)	65(0.98)	12(0.26)	274(3.78)
married	164(1.64)	43(0.65)	26(0.56)	233(3.22)
others	135(1.35)	47(0.71)	11(0.23)	193(2.66)

**Educational level**				
illiterate/elementary school	76(0.76)	40(0.60)	20(0.43)	136(1.88)
guidance/high-school	188(1.88)	62(0.93)	16(0.34)	266(3.67)
diploma/associate degrees	198(1.98)	47(0.71)	13(0.28)	258(3.56)
bachelor/higher	34(0.34)	6(0.09)	0(0)	40(0.55)

**Age (yrs)**				
15–30	288(2.89)	83(1.25)	17(0.37)	388(5.36)
31–39	73(0.73)	25(.037)	21(0.45)	119(1.64)
over 40	135(1.35)	47(0.71)	11(0.23)	193(2.66)

Table 3 summarizes demographic and socio-economic characteristics of subjects who scored ≥ 20. There were significant differences between depressive symptoms with the variables such as gender, age, socio-economic class, marital status and educational level. Socio-economic class had the highest difference between them. (P < 0.001).

**Table 3:** Demographic and socio-economic characteristics of subjects with BDI score ≥ 20

Variables	(BDI score ≥ 20) depressive symptoms		
	
	No	%	p-value
**Gender**			<0.001
female	461	65.85	
male	239	34.15	

**Socio-economic class (score)**			<0.001
high-class (19–25)	2	0.28	
middle-class (12–18)	87	12.43	
low-class (5–11)	611	87.29	

**Marital status**			<0.01
single	274	39.15	
married	235	33.57	
others	193	27.58	

**Educational level**			<0.001
illiterate/elementary school	136	19.42	
guidance/high-school	266	38	
diploma/associate degrees	258	36.86	
bachelor/higher	40	5.72	

**Age (yrs)**			<0.001
15–30	388	55.42	
31–39	119	17	
over 40	193	27.58	

Demographic and socio-economic characteristics of under study population according to the presence of depressive disorders in clinical interview are shown in Table 4. Again, there was a significant difference between presence of depressive disorders and mentioned variables and socio-economic class was of the most significant difference. (P < 0.001).

**Table 4:** Demographic and socio-economic characteristics of subjects with depressive disorders in psychiatric interview

Variables	Depressive disorders		
	
	No	%	p-value
**Gender**			<0.001
female	40	67.79	
male	19	32.21	

**Socio-economic class (score)**			<0.001
high-class (19–25)	1	1.69	
middle-class (12–18)	15	25.42	
low-class (5–11)	43	72.89	

**Marital status**			<0.001
single	28	47.45	
married	27	45.77	
others	4	6.78	

**Educational level**			<0.001
illiterate/elementary school	15	25.42	
guidance/high-school	24	40.67	
diploma/associate degrees	17	28.82	
bachelor/higher	3	5.1	

**Age (yrs)**			<0.030
15–30	33	55.93	
31–39	10	16.94	
over 40	16	27.11	

## Discussion

The aim of this study was to determine the prevalence of depressive disorders and some associated factors in Rasht City.

The results of our study indicated that 9.7% of subjects had depressive disorders which is consistent with the results of some studies [2,3] while it is different from another one [4].

These differences may be due to factors such as different tools and differences in method of assessment, sample size, age groups and definition of depressive disorders. In our study, prevalence rate of major depressive disorder was in concordance with Mohammadi et al study [4] while it was not supported by other studies [2,3,5]. We concluded that 2.5% and 5% of our samples were suffering from dysthymia and minor depressive disorder respectively which was different from Ayusa and Noorbala's findings [2-5].

Our study limitations in addition to higher participation of younger people than other age groups and possibly our weakness to distinguish minor depressive disorder from culture-bound mood experience, may explain aforementioned differences.

Our results indicated that the gender ratio (F: M) was approximately (2:1). This finding was confirmed by some studies [2-4] and opposed Stordal research [6].

Besides genetic differences between men and women, our developing society has radically changed the shape and content of women's lives. Participation of married women with children as a part of work force has become the norm and double shift is experienced by many women who provide home making and child care as well as working outside. Although women have benefited from changes in workplace and family roles, concomitant stress which results in increased level of depression is probably a consequence of double shift experience.

We concluded that depressive disorders were more common in people aged 15 to 30 which was different from other studies [3,4,6]. It may be due to higher participation of younger people and the higher prevalence of minor depressive disorder and dysthymia in comparison with major depressive disorder in this group in our study.

Results of this study showed that depressive disorders were more prevalent in singles and married people than others. In Vincinte and Stordal's study, the disorder was more common in singles and divorced subjects and less common in married [3,6]. By contrast, Mohammadi proved that depressive disorders were more common in married individuals in Iran [4]. We assumed that in our society, acceptance of divorce especially for women has negative social repercussions which may describe the higher prevalence of depressive disorders in married people.

Depressive disorders were of the highest prevalence in subjects with high-school and associate degrees educational level. Although this finding was approved in a study [8], Mohammadi and Vicente found different results [3,4]. It seems that unmet expectations of highly-educated people may explain this higher prevalence.

Our finding was similar to some previous studies [3,8] in the term of socio-economic status and was not compatible with the similar study by Mohammadi [4]. This difference might be due to factors such as, low participation of wealthy people, using different kind of socio-economic classification, and insufficient social support systems in under study society.

*Limitations of the present study: *1 – In the first phase, the proportion of male participated in the study, did not represent the actual male: female ratio in the corresponding society. 2 – In the second phase, an inadequate number of samples participated in clinical interview. 3 – The existence of up to 3 month interval between first and second phase of the study could be a potential source of bias as individuals might not accurately recall past symptomatology. 4 – Our descriptive research methodology only indicated point affective situation rather than its trends in the past and future and could not establish a relationship between background factors and studied variables. All of these limitations could result in an under or over – representation of prevalence rates in samples.

## Conclusion

Our study indicates that depressive disorder is common in Rasht City and it has different characteristics compared to other studies' findings especially Western studies. The prevalence of dysthymia and minor depressive disorder are more than major depressive disorder. Low socioeconomic class is the most significant factor associated with depressive symptoms (BDI) and depressive disorders (clinical interview).

Regarding our study limitations, researchers and policy makers should not consider our results as conclusive findings. We hope that the results of this survey will be used by researchers using analytical methodology to assess relationship between depressive disorders and associated factors.

## Authors' contributions

MJM: conceived of the study and participated in the design and co-ordination. HST: participated in the design of the study and interpretation of data. MF: participated in data collection and performed the statistical analysis. MS: participated in interpretation of data and drafting the article. AHM: participated in interpretation of data and drafting the article. All of the authors read and approved the final manuscript.
